# Appendicitis Complicating Pregnacy

**Published:** 1919-01

**Authors:** Aime Paul Heimeck

**Affiliations:** Chicago, Ill.


### Appendicitis Complicating Pregnancy.

By Aime Paul M. D., Chicago, Ill.

A review of the French, English and German literature on the subject of appendicitis complicating pregnancy, supplemented by my personal clinical experience, justifies the following conclusions:

1. Appendicitis occurs at all ages and in both sexes. Its frequency is underestimated and its significance not fully appreciated. It presents to all medical men important diagnostic, prognostic and therapeutic features.

2. Appendicitis, acute or chronic, initial, relapsing or recurrent, primary or secondary, complicates pregnancy with greater frequency than is believed. It is often overlooked, misdiagnosed and therefore improperly treated. It is the most important complication of pregnancy.

3. It occurs in single and twin gestations; in first, early and late pregnancies; in primiparae, deutiparae, and multiparae.

4. It occurs at all periods of the child-bearing age and at all periods of gestation. It complicates both intra- and extra-uterine pegnancies and can co-exist with other disease processes to which it may be primary, secondary or coincidental.

5. Gestation exerts no untoward influence upon the normal appendix. It can and frequently does aggravate existing, or determine new inflammatory disturbances in appendices deviating from the normal in form, length, mobility, location, etc., in appendices, the seat of inflammatory or other degenerative change or bound down by adhesions, or containing foreign bodies. Pregnancy does not relieve the dangers of appendicitis, but aggravates them.

6. Appendicitis and uni-or bilateral tubal pregnancy are frequently mistaken for each other. They may occur simultaneously or consecutively, may be either primary or secondary to, or independent of each other. Appendicitis may hasten tubal abortion through local infection, through general intoxication, may lead to suppuration of hematoceles, of fetal cysts.

7. In appendicitis, in ectopic pregnancy and in combined appendicitis and ectopic pregnancy, of obscure symptomatology, it matters not whether you are certain or in doubt as to the real diagnosis, early and timely operative treatment is imperatively indicated. Some cases of appendicitis in which extra-uterine pregnancy was thought to co-exist proved to be cases of appendicitis complicating uterine pregnancy.

8. During gestation, every type of appendicitis may occur: adhesive, catarrhal, gangrenous, ulcerative, obliterative, perforative, and suppurative. Pus may be present within the cavity of the appendix, in its wall or around it. An appendiceal or peri-appendiceal abscess may rupture spontaneously into the uterus, vagina, rectum, through the abdominal wall and into the peritoneal cavity.

9. Appendicitis with adhesion formation is of great significance, because adhesions of inflammatory origin can: (a) incarcerate the pregnant uterus in the pelvis and mechanically hinder the enlargement of the uterus; (b) impair the contractability of the uterus; (c) interfere with uterine labor contractions; (d) entail subinvolution; (e) induce sterility; (f) disturb tubal and varian integrity of function and of structure; (g) determine ileus; (h) produce abortion; and (i) lead to extra-uterine pregnancy.

10. Infection can and does spread from the appendix to the genital organs by way: (1) of the peritoneum (localized or diffuse peritonitis): (2) of the appendiculo-ovarian ligament; (3) of adhesions existing between the uterus and a perityphlitic pus focus; (4) of the Fallopian tube.

11. Simultaneous, consecutive, coincidental, inflammatory or other degenerative processes of the uterus, tubes or other pelvic organs may co-exist with appendicitis. The close anatomical relations existing between the appendix and the pelvic organs explain their frequent association in disease processes. It is easy to understand how inflammation can migrate from the appendix to the Fallopian tube, to the pregnant uterus, etc.

12. Appendicitis has a greater morbidity and a higher mortality in the pregnant than in the non-pregnant, operated or non-operated.

13. Appendicitis may or may not terminate pregnancy. The prognosis is good as to non-interruption of pregnancy: 1, when the appendix does not hang in the small pelvis; 2, when the inflammation is limited to the appendiceal mucosa; 3, when it does not extend beyond the appendiceal wall; 4, when the appendiceal abcess or peri-appendiceal abscess is small.

14. Pregnancy is a serious complication of appendicitis: 1, when the appendix is adherent to the uterus; 2, when it is the seat of an inflammation perforative, gangrenous or suppurative in type; 3, when its inflammation leads to abscess formation, near or distal; 4, when the uterus forms part of the wall of an appendicular, peri- or para-appendicular abscess. In the aforementioned conditions, adhesions may be torn, abscesses may be ruptured by the enlarging uterus.

15. The symptomatology of appendicitis in the pregnant is the same as in the non-pregnant. The clinical picture, however, is blurred by the co-existing symptoms of pregnancy. Mistakes are less likely to occur by keeping in mind that: (a) Every pregnant woman is to be examined for physical defects; (b) The history is all-important; ask about previous attacks. In cases of relapsing appendicitis in young women, the attacks frequently occur just before or at the menstrual period. (c) In gravid women, all attacks of indigestion associated with vomiting and fever should arouse suspicion and command a careful examination of the abdomen; (d) Right-sided iliac pain unassociated with uterine contractions should lead one to think of appendicitis. (e) Deep-seated retro-cecal and other abcesses may be detected by rectal examination. (f) Peri-and paratyphlitic abscesses may be detected by vaginal examination. Pregnancy increases the severity and the mortality of appendicitis.

16. The morbidity and mortality of appendicitis complicating pregnancy and the puerperium are the morbidity and mortality of delay in applying efficient surgical treatment. The initial symptoms of the attack do not enable the clinician to foretell accurately how a given case will terminate. What is going to happen in ten, twenty or forty hours following the onset of appendicitis can not be foreseen. When the condition is diagnosed and remedied early, the mortality is practically nil. Abscess formation may be forestalled by early diagnosis and early operation. The high mortality is due to late diagnosis and late operation. The pregnant woman whose metabolism is good is a good subject for operative measures.

17. The type and the acuity of the inflammation influence the prognosis. The prognosis is good if the changes in the appendix are slight; if the inflammation is limited to the appendiceal wall; if there be slight or no peritoneal involvement, if complications be absent. It is grave in gangrenous, perforative and suppurative appendicitis and in all cases complicated by abscess formation near or distal, or by diffuse peritonitis.

18. Prognosis is better for the mother if there be no interruption of pregnancy, spontaneous or otherwise. The bad attacks cause abortions and abortion aggravates the illness. In the great majority of surgically treated cases, there is no interruption of pregnancy, and when it does occur it is not due directly to the operation. The interruption of pregnancy is not indicated. It aggravates the prognosis.

19. The results for the mother and fetus are better, the less advanced the gestation, the less virulent and widespread the inflammation, the earlier the operation.

20. As far as the child is concerned, prognosis is absolutely good in cases of early operated appendicitis. Severe maternal appendicitis is exceptionally grave for the fetus, who succumbs either through infection or through interruption of pregnancy.

21. The following prophylactic measures are sound and safe and are recommended for general adoption: (a) During the child-bearing age, recurrent attacks of pelvic pains, dysmenorrhea, menstrual and other pelvic disturbances unassociated with objective pelvic findings are not infrequently due to unrecognized appendicitis or sequelae thereof. In the presence of this etiological factor, the ablation of the appendix is indicated. (b) In laparotomies for conditions other than appendicitis, the appendix should be examined. Should it present any deviation from the normal, its removal is indicated. (c) During the child-bearing age, any woman who has had one or more attacks of appendicitis treated non-operatively should have her appendix removed so as to correct existing pathological conditions and prevent future attacks of appendicitis and complications thereto. True prophylaxis in a woman of child-bearing age who has had one or more well-marked attacks of appendicitis is an interval operation. It goes without saying that constipation is to be avoided and that other hygienic precautions are to be observed. A definite and accurate diagnosis of acute, chronic or recurrent appendicitis invariably calls for operation, irrespective of the stage of pregnancy. The disease, during pregnancy, runs such a rapid destructive course that delay is hazardous. Operation should be early and immediate. A case may be rendered hopeless by hesitation and inaction. Temporizing methods are extremely dangerous.

22. Treat appendicitis in the pregnant female as you treat it in the non-pregnant. Every pregnant woman who is a subject of appendicitis should be operated on just as soon as the diagnosis is made, whether the attack is the first, second or third.

The unusual risks of leaving a diseased appendix in the abdominal cavity are much increased by the pregnant state and the evil consequences of another attack, i. e., gangrene or perforation, will be correspondingly greater. The danger of recurrence in the later months of pregnancy and in the child-bed calls for operation, preferably during the attack. If the patient is not seen in time, one will do the next best thing, an interval operation during the pregnancy. Pregnancy is an additional indication for operation in cases of appendicitis.

23. In inflammatory disease of the appendix, the ideal operation is appendectomy. In some cases, however, one has to be content with incision, evacuation and drainage of an appendiceal abscess. Exceptionally, drainage of abscess in Douglas' pouch may be effected through the vagina or rectum. Pus should be evacuated, irrespective of uterine contents and irrespective of its location.

24. It is well to keep in mind that for an appendectomy the median incision is contra-indicated in the later months of pregnancy, that it is best to avoid or to reduce to a minimum, the manipulations of the uterus; opiates are indicated in the after treatment. Labor when it occurs shortly after a laparotomy is not to be unduly prolonged; it may have to be assisted. When operating for appendicitis in a pregnant woman, every effort should be put forth to prevent miscarriage. Interruption of pregnancy is not indicated as it increased the danger.

25. In all laparotomies for conditions other than appendicitis, if the patient's condition permits, the appendix should be examined and removed: 1, if it be abnormal in length, size or location; 2, if it be in close relation to a pedicle or denuded surface, left by operation; 3, if its cavity be partly or wholly obliterated; 4, if it be the seat of anatomic alterations, club-shaped, thickened, kinked, twisted, strictured, etc.; 5, if it contains foreign bodies, fecal concretions, worms, etc.; 6, if it be adherent, in part or in its entirety, to some normal or diseased contiguous organ or to the abdominal parietes; 7, if it be the sole content or one of the contents of a hernial sac; 8, if it be the seat of cystic, neoplastic or inflammatory disease.

26. When in doubt as to whether the case is one of appendicitis, salpingitis, tubal pregnancy or other pathological conditions, use a supra-pubic median incision. This incision affords easy acess to most of the pelvic contents and though it is not the incision of election for exposure of the appendix, it is a very serviceable incision.

